# Over-Expression of *OsHOX24* Confers Enhanced Susceptibility to Abiotic Stresses in Transgenic Rice via Modulating Stress-Responsive Gene Expression

**DOI:** 10.3389/fpls.2017.00628

**Published:** 2017-04-21

**Authors:** Annapurna Bhattacharjee, Raghvendra Sharma, Mukesh Jain

**Affiliations:** ^1^National Institute of Plant Genome ResearchNew Delhi, India; ^2^School of Computational and Integrative Sciences, Jawaharlal Nehru UniversityNew Delhi, India

**Keywords:** abiotic stress, homeobox, microarray, over-expression, regulation, rice, transcription factor, transgenics

## Abstract

Homeobox transcription factors play critical roles in plant development and abiotic stress responses. In the present study, we raised rice transgenics over-expressing stress-responsive *OsHOX24* gene (rice homeodomain-leucine zipper I sub-family member) and analyzed their response to various abiotic stresses at different stages of development. At the seed germination stage, rice transgenics over-expressing *OsHOX24* exhibited enhanced sensitivity to abiotic stress conditions and abscisic acid as compared to wild-type (WT). *OsHOX24* over-expression rice seedlings showed reduced root and shoot growth under salinity and desiccation stress (DS) conditions. Various physiological and phenotypic assays confirmed higher susceptibility of rice transgenics toward abiotic stresses as compared to WT at mature and reproductive stages of rice development too. Global gene expression profiling revealed differential regulation of several genes in the transgenic plants under control and DS conditions. Many of these differentially expressed genes were found to be involved in transcriptional regulatory activities, besides carbohydrate, nucleic acid and lipid metabolic processes and response to abiotic stress and hormones. Taken together, our findings highlighted the role of OsHOX24 in regulation of abiotic stress responses via modulating the expression of stress-responsive genes in rice.

## Introduction

Extreme environmental perturbations, such as drought, cold, high salinity and temperature influence the growth, survival and productivity of plants. The economically important cereal crops like rice are severely affected due to the adverse environmental onslaughts leading to heavy losses in yield. During abiotic stress conditions, the transcript levels of various stress-responsive genes are altered in plants. Several transcription factors (TFs) are known to be prominently involved in abiotic stress responses. They are major components of transcriptional regulatory networks called regulons as they modulate the expression of several downstream target genes during abiotic stresses in plants (Nakashima et al., 2009; Urano et al., 2010; Todaka et al., 2015). TFs have been used as potent tools to engineer abiotic stress tolerance in plants (Golldack et al., 2011; Wang et al., 2016). Over-expression of several abiotic stress-responsive TFs, like DREBs, AREBs and NACs led to the generation of stress-tolerant transgenic plants without loss in crop yield (Nakashima et al., 2009; Todaka et al., 2015). However, function of most of the TFs still remains unexplored in context of abiotic stress tolerance.

Homeobox TFs are known to play an integral role in crucial developmental processes in plants and have been found to be differentially expressed under abiotic stress conditions in various crop species. Some of them have been characterized via transgenic/mutant analysis in model plants (Deng et al., 2002; Olsson et al., 2004; Jain et al., 2008; Yu et al., 2008, 2013; Mukherjee et al., 2009; Zhao et al., 2011; Zhang et al., 2012; Chew et al., 2013; Bhattacharjee et al., 2015, 2016). These evidences indicate that homeobox TFs can act as mediators of plant growth response under different abiotic stress conditions. Thus, homeobox TFs have been speculated to act as promising candidates for crop improvement (Zhang et al., 2012; Bhattacharjee and Jain, 2013; Chew et al., 2013; Yu et al., 2013). However, only few studies have explored the role of rice homeobox TFs in abiotic stress responses so far (Luo et al., 2005; Zhang et al., 2012) and functional characterization of most members of rice homeodomain-leucine zipper I (HD-ZIP I) subfamily remains to be carried out as of now. Interestingly, interaction of homeobox TFs with members belonging to same family and other proteins have also been reported (Meijer et al., 2000; Johannesson et al., 2001; Deng et al., 2002; Tran et al., 2007; Bhattacharjee et al., 2016). Further, DNA binding properties of homeobox TFs have also been explored to some extent (Sessa et al., 1997; Palena et al., 1999; Meijer et al., 2000). In addition, some studies have reported the identification of few downstream target genes of homeobox TFs (Deng et al., 2006; Manavella et al., 2006; Ariel et al., 2010). However, the exact regulatory role of homeobox TFs in abiotic stress responses has not been deciphered till now.

In our previous study, we showed differential regulation of *OsHOX24* under abiotic stress conditions in rice and also demonstrated that its over-expression imparts higher sensitivity to abiotic stresses in transgenic Arabidopsis (Bhattacharjee et al., 2016). Here, we further characterized the function of *OsHOX24* by raising over-expression transgenic rice plants. We assessed the rice transgenics under various abiotic stress conditions at different developmental stages. In addition, global transcript profiling enabled us to recognize diverse downstream target genes of OsHOX24. Our investigation demonstrated the role of *OsHOX24* in abiotic stress responses via controlling stress-responsive gene expression.

## Materials and Methods

### Generation of Rice Transgenics

To over-express *OsHOX24* in rice, PCR amplified complete open reading frame (ORF) [using gene specific primers (Supplementary Table S1)], was cloned in modified pCAMBIA1301 vector in *Bam*HI/*Kpn*I restriction sites. The confirmed clone was transformed in *Agrobacterium* strain LBA4404. Rice seeds [*Oryza sativa* cultivar Pusa Basmati 1 (PB1)] were used as background for generation of transgenic plants. The transformation of embryogenic calli derived from the scutellum of dehusked mature rice seeds was done as described previously (Sharma et al., 2013). Rice transformants were confirmed by Southern blotting. Seeds obtained from T1 generation plants were screened on Murashige–Skoog (MS) media supplemented with hygromycin (40 mg/L). Further, segregation ratio of each confirmed transgenic line was estimated and transgenics were grown till homozygous stage to obtain seeds for future analyses.

### Phenotypic Assays

To study the effect of transgene in over-expression rice transgenics, phenotypic characterization of wild-type (WT) and rice transgenics over-expressing *OsHOX24* was carried out. Three-week-old rice seedlings grown in the culture room, were transferred to pots and grown till maturity with optimum supply of water. Growth of plants was monitored and several phenotypic parameters, like shoot length, flag-leaf area, number of panicles and tillers per plant were documented in three independent biological replicates, consisting of at least 13–15 plants per line.

### Seed Germination and Stomatal Opening/Closure Assays

To assess the performance of rice transgenics under abiotic stress conditions, seed germination assays were carried out. Seeds from transgenic and WT plants were plated on MS medium without or with ABA (5 and 10 μM), 200 mM NaCl, 200 mM mannitol and -0.4 MPa PEG 6000. Seed germination was recorded after 3 days of transferring the plated seeds to culture room except for PEG 6000 treatment (germination was recorded after 7 days). The number of germinated seeds (considering radicle emergence as seed germination parameter) was expressed as percentage of total number of seeds plated, as described earlier (Dansana et al., 2014). Each experiment was repeated at least three times.

For stomatal opening/closure assays, 7-day-old rice seedlings were kept in Yoshida medium (experimental control) and subjected to exogenous ABA treatment (100 μM) for 3 h. After incubation, leaf sections were visualized under scanning electron microscope (Zeiss EVO LS10, Germany) and stomatal status (opened/partially opened/closed) were analyzed for each sample. The experiment was repeated at least three times.

### Evaluation of Plants under Abiotic Stress Conditions

For evaluating the effect of salinity and desiccation stresses (DSs) on seedlings, WT and *OsHOX24* rice transgenics (H1, H49 and H74) were grown on MS medium supplemented without or with NaCl (200 mM) and PEG 6000 (20%) in the culture room for 12 days. The root and shoot lengths of seedlings grown under control and stress conditions were measured. The relative average root and shoot lengths under salinity and DS conditions were expressed as percentage of root and shoot lengths of the seedlings under control condition. The experiments were performed in at least three independent biological replicates.

To assess the effect of DS, rice transgenics and WT plants, grown in greenhouse for 2 months till mature vegetative stage (6 weeks) and reproductive stage (15 weeks) were subjected to DS by withholding water for 5 weeks, followed by 2 weeks of recovery. WT and transgenic plants of same age served as experimental controls. Plant growth was monitored at all stages and phenotype of plants under control and DS followed by recovery phase was documented. To determine the effect of DS, chlorophyll content in transgenic and WT leaves under desiccation and control conditions at mature and reproductive stages after recovery phase, was estimated using a Chlorophyll Meter (SPAD-502, Japan). To assess the effect of DS, number of turgid leaves was counted and survival percentage of plants was calculated after recovery. In addition, rate of water loss in transgenic and WT leaves at mature and reproductive stages were estimated as described (Dansana et al., 2014). The experiments were performed in at least two independent biological replicates.

### Leaf Disk Assays

To determine the effect of DS on rice transgenics and WT, leaf disk assays were carried out. Healthy and fully expanded leaves from 2-month-old WT and rice transgenics were detached and 4–5 leaf disks were floated in sterile water (experimental control) or 20% PEG 6000 solution and samples were incubated under culture room conditions for 3 days. The effect of DS was assessed by monitoring phenotypic changes and measuring chlorophyll content of leaves relative to control samples. Chlorophyll was extracted from leaf samples and the amount of chlorophyll a, chlorophyll b and total chlorophyll was calculated as described previously (Sharma et al., 2013).

### Statistical Analysis

All the experiments were performed in at least two or three independent biological replicates and standard error (SE) was computed. For estimation of statistical significance, Student’s *t-*test was performed. Statistically significant differences between WT and transgenics or between control and stress conditions (^∗^*P* ≤ 0.05 and ^∗∗^*P* ≤ 0.01) were denoted by asterisks.

### Microarray Analysis of Rice Transgenics

Total RNA isolated from 3-week-old transgenic and WT seedlings subjected to 3 h of control and DS treatment were used for microarray analysis and quality control of samples was carried out as described (Sharma et al., 2013). Microarray analysis for rice seedlings was performed using Affymetrix WT PLUS Reagent kit (Affymetrix, Santa Clara, CA, USA) in three independent biological replicates, according to manufacturer’s instructions. Normalization of microarray data was done by Robust Multi-array Average (RMA) algorithm implemented in Genespring software version 12.6. The microarray data has been submitted in the Gene Expression Omnibus database at NCBI under series accession number GSE79212. Differential gene expression analysis was performed as described earlier (Sharma et al., 2013).

Gene ontology (GO) enrichment was performed using BiNGO. The stress response pathway analysis was carried out using MapMan (version 3.5.1)^1^ with *P*-value cut-off of ≤0.05. Venn diagrams and heatmaps were generated using online tools, VENNY^2^ and MeV (version 4.9), respectively.

### Quantitative Reverse Transcription Polymerase Chain Reaction (qRT-PCR) Analysis

For gene expression profiling, total RNA isolation, first-strand cDNA synthesis and qRT-PCR analysis were performed as described previously (Sharma et al., 2013). Experiments were conducted in at least two biological replicates for each sample and three technical replicates were analyzed for each biological replicate. ΔΔC_T_ calculation method was used to calculate relative expression level of each gene. For normalizing the relative mRNA level of individual gene in various RNA samples, *Ubiquitin 5* (*UBQ5*) was used as internal control gene (Jain et al., 2006). Results of microarray experiments were validated by qRT-PCR analysis of selected differentially expressed genes. The list of primer sequences used for qRT-PCR analysis has been provided in Supplementary Table S1.

## Results

### Generation of Transgenic Rice Plants Over-expressing *OsHOX24*

**FIGURE 1:**
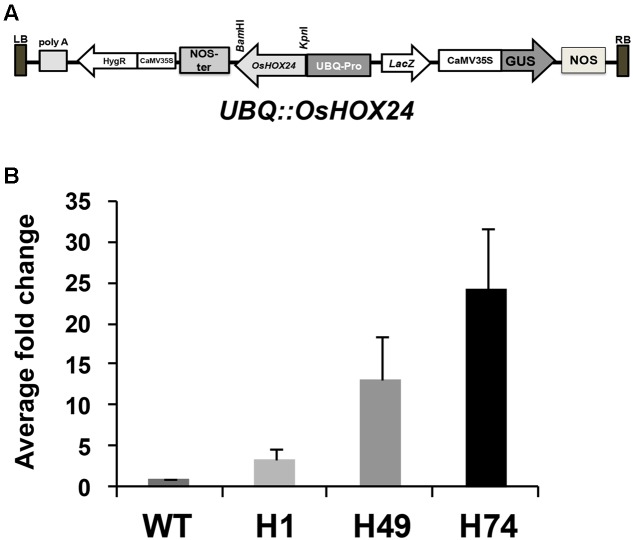
**Over-expression of *OsHOX24* in rice.** **(A)** Schematic representation of *OsHOX24* over-expression construct used for raising rice transgenics. **(B)** Relative expression profiles of *OsHOX24* in the rice transgenic lines (H1, H49 and H74) as compared to wild-type (WT) are shown. Values are mean (*N* = 3) from three independent experiments. Error bars indicate SE.

The complete coding region of *OsHOX24* was over-expressed under the control of *ubiquitin (UBQ)* promoter (*UBQ::OsHOX24*) in rice (**Figure 1A**). A total of 18 hygromycin-resistant T0 transgenic plants were obtained. Three of these lines, exhibiting 3:1 segregation ratio in T1 generation and confirmed by Southern blotting, were used for further analyses. The enhanced expression level of transgene in all the selected transgenic lines as compared to WT was detected by real-time PCR analysis (**Figure 1B**). The relative expression level of *OsHOX24* was found to be highest in H74 line followed by H49 and H1 lines.

We did not observe any detectable phenotypic differences in the *OsHOX24* rice transgenics as compared to WT at the seedling stage under control conditions (Supplementary Figure S1). However, over-expression of *OsHOX24* resulted in significant alteration in the phenotype of rice transgenics (as compared to WT) at the reproductive stage (Supplementary Figure S2A). Transgenic lines showed significantly reduced shoot length (87–89%) (Supplementary Figure S2B) and smaller flag-leaf area (61–81%) as compared to WT (Supplementary Figure S2C). Moreover, the number of panicles (Supplementary Figure S2D) and tillers (Supplementary Figure S2E) were also found to be lesser in the transgenic lines as compared to WT. In transgenic lines, panicle and tiller numbers ranged from 65–73% and 69–73%, respectively, as compared to WT (Supplementary Figures S2D,E). These observations indicated the role of *OsHOX24* in modulating plant phenotype during reproductive development in rice.

### Rice Transgenics Showed Reduced Germination and Impaired Stomatal Closure under Abiotic Stresses

**FIGURE 2:**
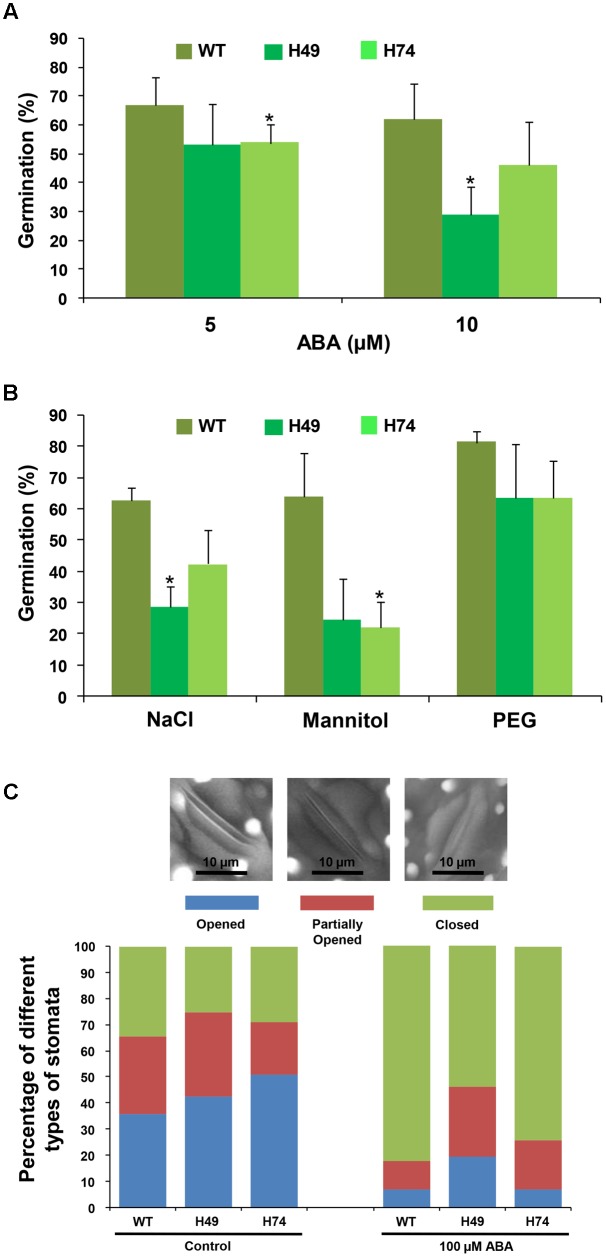
**Over-expression rice transgenics show higher sensitivity to abiotic stress conditions during seed germination and impaired stomatal closure under exogenous ABA treatment.** Effect of ABA **(A)** and abiotic stress treatments **(B)** [200 mM NaCl, 200 mM mannitol and (–0.4 MPa) PEG 6000] on seed germination of WT and rice transgenic lines (H49 and H74) is represented. In case of NaCl and mannitol, seed germination after 3 days have been reported, whereas germination of seeds on PEG 6000 recorded after 7 days, have been reported. The number of germinated seeds was expressed as the percentage of total number (15–20) of seeds plated. Values are mean (*N* = 3) from at least three independent experiments. Error bars indicate SE. Data point marked with asterisk (^∗^ *P* ≤ 0.05) indicate statistically significant difference. **(C)** Bar graph depicting the percentage of different stomata types [opened (OS), partially opened (POS) and closed (CS)] recorded in WT and rice transgenic lines under control condition and exogenous ABA treatment. At least 50 stomata were analyzed for each line under each condition. The representative images showing different stomata types (OS, POS and CS) are given above the bar graph.

To evaluate the effect of stress hormone, ABA and abiotic stress conditions (osmotic, salinity and desiccation) on the transgenic lines and WT, seed germination assays were carried out. The percentage germination of transgenic lines was much lesser as compared to WT on MS medium supplemented with different concentrations of ABA (5 and 10 μM), NaCl (200 mM), mannitol (200 mM) and PEG 6000 (-0.4 MPa) [equivalent to 20% PEG 6000] (**Figure 2**). Exogenous ABA treatment resulted in greater susceptibility of transgenics as compared to WT. For instance, transgenics showed only about 53% germination after 3 days on 5 μM ABA as compared to 66% for WT. At 10 μM ABA, WT showed 61% germination in comparison to 28–45% germination of transgenics (**Figure 2A**). Under salinity stress (200 mM), WT exhibited 62% germination, whereas transgenic lines exhibited significantly reduced germination (28–42%) (**Figure 2B**). Similarly, the effect of osmotic stress (200 mM mannitol) on germination was found to be more prominent on transgenics (**Figure 2B**), which showed only 22–24% germination in comparison to WT (63%). Under DS (20% PEG 6000 treatment), transgenic lines showed 63% germination as compared to 81% in WT (**Figure 2B**). Overall, these results indicated that *OsHOX24* over-expression altered seed germination in the transgenic plants under abiotic stress conditions. Among the transgenic lines, H49 exhibited greater susceptibility to different abiotic stresses.

Further, we investigated the effect of ABA on stomatal closure in leaves of transgenics and WT. Under control conditions, transgenics and WT showed similar number of opened and closed stomata. However, the fraction of completely closed stomata in transgenics was considerably lesser (53–74%) as compared to WT (82%) under exogenous ABA treatment (**Figure 2C**). This observation indicated that transgenics possess significantly reduced ability of stomatal closure under stress condition.

### Rice Transgenics Exhibited Greater Susceptibility to Abiotic Stresses at the Seedling Stage

**FIGURE 3:**
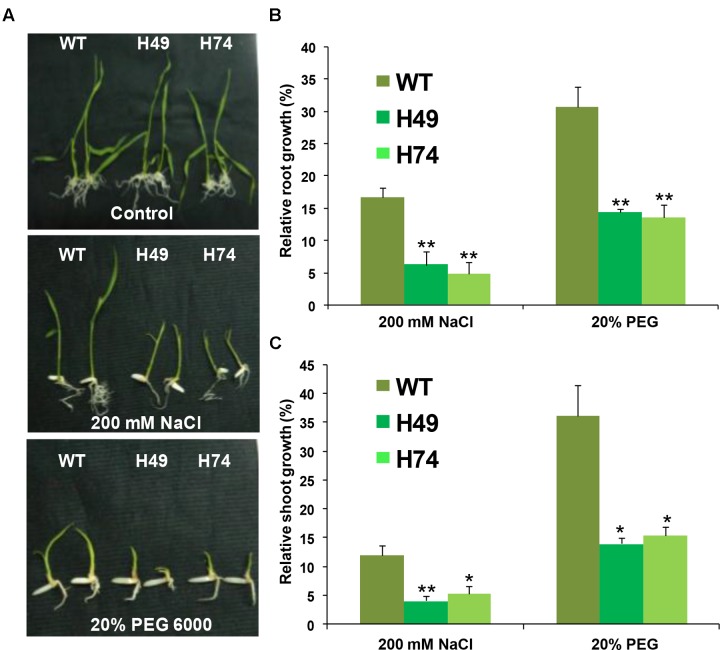
**Over-expression rice transgenic lines show lesser growth as compared to WT plants under abiotic stress conditions at seedling stage.** **(A)** Phenotypes of 12-day-old WT and rice transgenic seedlings (H49 and H74) under control and abiotic stress conditions are shown. Effect of 200 mM NaCl and (–0.4 MPa) PEG 6000 treatment (20% PEG) on root **(B)** and shoot **(C)** growth of WT and transgenic lines are graphically represented. The relative average root **(B)** and shoot **(C)** growth of seedlings grown in abiotic stress conditions were expressed as percentage of average root and shoot growth of seedlings grown on MS medium under control condition. Experiments were performed in three independent biological replicates. Values shown in graphs are mean from a single representative biological replicate (*n* = 15–20). Error bars indicate SE. Bars marked with asterisk (^∗^ *P* ≤ 0.05, ^∗∗^ *P* ≤ 0.01) indicate statistically significant difference.

The effect of desiccation and salinity stresses on growth of transgenic and WT seedlings were evaluated. A significant reduction in root and shoot growth was observed in transgenics as compared to WT seedlings under salinity (200 mM NaCl) and desiccation (20% PEG 6000) stresses, whereas all the seedlings appeared healthy under control conditions (**Figure 3A**). The average root growth of transgenics was found to be 4–8% as compared to 16% for WT under salinity stress relative to control condition. Similarly, average root growth of transgenics was found to be 13–14% as compared to 30% in WT under DS (**Figure 3B**). The average shoot growth of transgenics was 4–5% as compared to 12% in WT under salinity stress, and 14–15% as compared to 36% in WT under DS (**Figure 3C**).

### Rice Transgenics Displayed More Susceptibility to Abiotic Stress Conditions at Mature and Reproductive Stages of Development

Over-expression of *OsHOX24* affected the rate of water loss in transgenic rice. The transgenic leaves exhibited comparatively greater water loss at mature and reproductive stages of development than WT till 210 min of air-drying (Supplementary Figure S3). Detached leaves of WT from the mature and reproductive stage of development retained 69 and 60% of fresh weight as compared to 46 and 47% of fresh weight retained by the transgenic line (H49) following 90 min of air drying, respectively (Supplementary Figure S3). After 210 min of air drying, detached WT leaves taken at the mature and reproductive stage of development retained 34–40% of fresh weight, in contrast to transgenic line (H49) which retained only 26–30% of fresh weight (Supplementary Figure S3).

**FIGURE 4:**
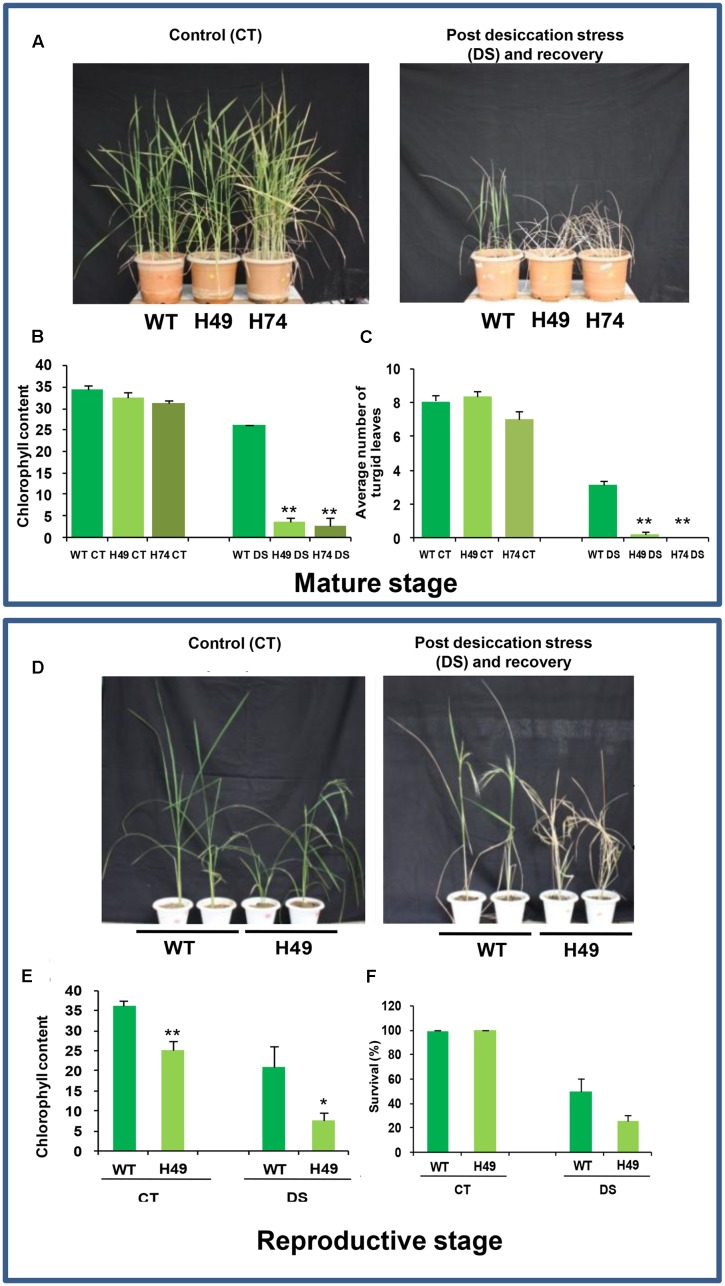
**Over-expression rice transgenics are more susceptible to desiccation stress (DS) as compared to WT plants at mature and reproductive stages of development.** **(A)** Phenotype of 110-day-old WT and rice transgenic (H49 and H74) plants under control condition (Left) and post DS and recovery (Right). Effect of DS on WT and transgenics on chlorophyll content **(B)** and number of turgid leaves **(C)**. **(D)** Phenotype of 150-day-old WT and transgenic (H49) plants under control condition (Left), and post DS and recovery (Right). Effect of DS on WT and transgenics on chlorophyll content **(E)** and survival percentage **(F)**. The experiments were conducted in at least two independent biological replicates. Values are mean from one representative biological replicate (*n* = 10–15). Chlorophyll content was measured in SPAD units. Error bars indicate SE. Data points marked with asterisk (^∗^*P* ≤ 0.05; ^∗∗^ *P* ≤ 0.01) indicate statistically significant difference.

To assess the effect of DS at mature vegetative stage, leaf disk assays were performed for transgenics and WT plants using 20% PEG 6000. The transgenic leaves exhibited greater chlorophyll loss as compared to WT (Supplementary Figure S4). The chlorophyll content of transgenic lines, H49 (60%) and H74 (61%), was lesser in comparison to WT (86%) under DS relative to control condition (Supplementary Figure S4). Further, 2-month-old *UBQ::OsHOX24* transgenic lines and WT were subjected to DS by withholding water for 5 weeks followed by recovery for 15 days. The transgenic lines showed greater wilting of leaves and poor recovery as compared to WT (**Figure 4A**). The extent of chlorosis was more prominent in the leaves of transgenic lines as compared to WT (**Figure 4B**). The chlorophyll content in transgenic lines was significantly lesser than WT under DS (**Figure 4B**). The transgenic lines possessed lesser number of turgid leaves than WT under DS (**Figure 4C**). The above observations indicated the higher susceptibility of *OsHOX24* over-expressing transgenics under DS as compared to WT at the mature stage of development as well.

To evaluate the effect of DS at the reproductive stage, 15-week-old WT and *UBQ::OsHOX24* transgenic line (H49) were subjected to DS by withholding water for 30 days followed by recovery for 15 days. Transgenics (H49) showed more compromised growth, greater wilting of leaves and poor recovery than WT (**Figure 4D**). The actual chlorophyll content in the transgenic line was evidenced to be much lesser than WT under DS and control condition (**Figure 4E**). The chlorophyll content of transgenic line was only 35% as compared to WT under DS (**Figure 4F**). In addition, under control condition too, the chlorophyll content of transgenic line was lesser (only 69%) in comparison to WT (**Figure 4E**). In addition, the transgenic line exhibited lower survival percentage as compared to WT (**Figure 4F**). Only 25% of the transgenic plants survived as compared to WT (50%) under DS (**Figure 4F**). The above observations confirmed the susceptible nature of *UBQ::OsHOX24* transgenics under DS as compared to WT at the reproductive stage of development too.

### Global Gene Expression Profiling

**FIGURE 5:**
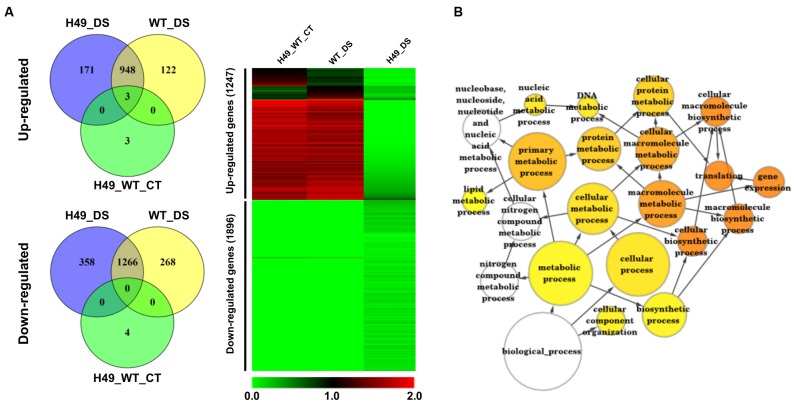
**Differential gene expression in *OsHOX24* over-expression transgenic (H49) as compared to WT under control and DS.** **(A)** Venn diagrams (Left) representing distribution of up-regulated and down-regulated genes in different conditions. Heat-map (Right) representing expression profiles of differentially expressed genes in H49_WT_CT (differential expression between WT and H49 under control condition), WT_DS (differential expression in WT under DS) and H49_DS (differential expression in H49 under DS) conditions. The average log signal values are shown by color scale. **(B)** The significantly enriched gene ontology (GO) categories in the down-regulated genes in H49. Node size is proportional to the number of genes. Color shading is given according to *P*-value (white: no significant difference; yellow = 0.05, orange < 0.0000005).

**FIGURE 6:**
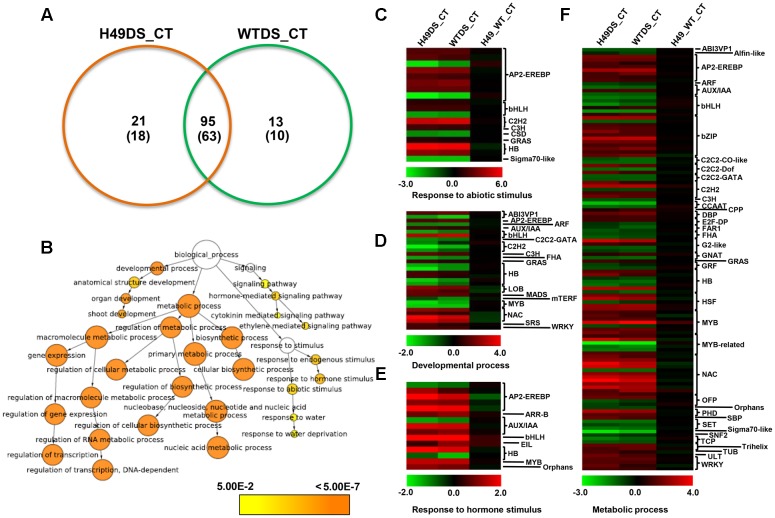
**Differential expression of transcription factor (TF)-encoding genes and functional categorization in *OsHOX24* transgenics.** **(A)** Venn diagram (Left) representing distribution of up-regulated and down-regulated (in parentheses) genes in transgenic (H49) and WT under DS. **(B)** The significantly enriched GO categories in the differentially expressed TF-encoding genes in the transgenic and WT under control and DS. The differentially expressed TF-encoding genes were analyzed using BiNGO. Node size is proportional to the number of genes. Color shading is given according to *P*-value (white: no significant difference; yellow = 0.05, orange < 0.0000005). **(C–F)** Heat-maps representing expression profiles of differentially expressed TF-encoding genes related to response to abiotic stimulus **(C)**, developmental process **(D)**, response to hormone stimulus **(E)** and metabolic process **(F)** in H49_WT_CT (differential expression between WT and H49 under control condition), WT_DS (differential expression in WT under desiccation and control condition) and H49_DS (differential expression in H49 under desiccation and control condition). The average log signal values are shown by color scale.

To study the effect of *OsHOX24* over-expression in rice, microarray analysis of transgenic line (H49) and WT plants, under control condition and subjected to DS treatment, was performed. The differential gene expression analysis, revealed a total of 3108 significantly (≥twofold, corrected *P*-value ≤ 0.05) differentially regulated genes in the transgenic line as compared to WT under control and/or DS. A total of 1247 and 1896 genes were found to be up-regulated and down-regulated, respectively, in at least one of the conditions analyzed (**Figure 5A** and Supplementary Table S2). These genes were found to be involved in diverse biological processes. GO enrichment of down-regulated genes in the transgenic line under DS revealed that several cellular metabolic and biosynthetic processes (primary metabolic processes and nucleic acid metabolic processes) were significantly enriched (**Figure 5B**). About 7% of the differentially expressed genes belonged to 51 different TF families (Supplementary Figure S5). At least 39 and 23 TF-encoding genes were uniquely differentially expressed in the transgenic line (H49) and WT, respectively, whereas 158 genes were found to be commonly differentially regulated in the transgenic line and WT under DS (**Figure 6A**). GO enrichment analysis of these TF-encoding genes revealed their involvement in crucial biological processes, including gene expression, developmental processes, metabolic processes, response to abiotic stress and hormone stimulus (**Figures 6B–F**). Further, biotic and abiotic stress response pathway overview indicated that genes involved in signaling pathways, hormone signaling (auxin, ABA, brassinosteroid and ethylene), stress-responsive transcription regulatory components (including TFs like ERF, bZIP, WRKY and MYB), redox metabolism and respiratory burst, defense responses, secondary metabolism, proteolysis and cell wall, were enriched in the transgenic line under DS (Supplementary Figure S6). This indicated the role of *OsHOX24* in biotic stress responses, besides mediating abiotic stress responses in transgenic rice.

Overall, the differentially expressed genes were found to be involved in diverse metabolic and developmental processes. The differential expression patterns of selected stress-responsive rice genes, known to be involved in abiotic stress responses, including *LOC_Os07g14610* encoding for IAA-amino acid hydrolase, *LOC_Os01g07120* encoding for OsDREB2A, *LOC_Os07g37400* encoding for OsFBX257, *LOC_Os05g37060* encoding for MYB TF and *LOC_Os12g03050* encoding for NAM TF were validated via real-time PCR analysis (Supplementary Figure S7). Overall, the transcriptome analysis of transgenic line and WT under control and DS revealed alteration of several developmentally important and stress-related genes, which explain the susceptible phenotype of rice transgenics as compared to WT.

## Discussion

Members of HD-ZIP superclass belonging to homeobox gene family are key plant-specific regulators of developmental program (Ariel et al., 2007; Chew et al., 2013; Turchi et al., 2015). Some of the HD-ZIP TFs have been implicated in abiotic stress responses as well (Olsson et al., 2004; Agalou et al., 2008; Jain et al., 2008; Yu et al., 2008; Song et al., 2012; Bhattacharjee et al., 2016). Previously, we found at least nine HD-ZIP I genes to be differentially expressed under various abiotic stress conditions in rice (Jain et al., 2008). However, functionality of very few rice HD-ZIP I subfamily members has been investigated *in planta* till now (Zhang et al., 2012; Bhattacharjee et al., 2016). Recently, we characterized two HD-ZIP I members, namely *OsHOX22* and *OsHOX24*, and suggested their role in abiotic stress responses via raising over-expression transgenics in model plant Arabidopsis (Bhattacharjee et al., 2016). It is likely that a gene shows similar function in different plant systems. However, there are some evidences where over-expression of the same gene has resulted in varied phenotypes of transgenics in diverse plant systems (Jang et al., 2007; Alavilli et al., 2016; Zheng et al., 2016).

In the present study, we carried out functional characterization of *OsHOX24* by raising over-expression transgenic lines in crop plant rice to ascertain its function in abiotic stress responses and prove its potential in engineering stress tolerance in crop plants as well. The transgenic rice over-expressing *OsHOX24* showed compromised phenotype in comparison to WT plants at reproductive stage of development, which suggested the role of *OsHOX24* as a potential developmental regulator in rice. However, its exact role in rice development needs to be explored further in more detail. Our observations corroborated the findings in previous reports, where over-expression of TF-encoding genes, *Oshox22* and *OsDof12* led to altered phenotype in rice transgenics at reproductive stage of development (Zhang et al., 2012; Wu et al., 2015). Seed germination and root/shoot growth assays, revealed the susceptible nature of rice transgenics at seedling stage under desiccation, salinity and osmotic stresses. Similar observations were witnessed when transgenic Arabidopsis over-expressing *OsHOX24* were analyzed vis-a-vis WT plants under abiotic stress conditions (Bhattacharjee et al., 2016). *OsHOX24* over-expression transgenic rice lines were more susceptible to abiotic stresses as compared to WT under DS treatment at mature and reproductive stages of development as well. In our previous study, transgenic Arabidopsis over-expressing *OsHOX24* were also found to be more susceptible to water-deficit stress as compared to WT plants at mature stage of development (Bhattacharjee et al., 2016). Similar results were observed when *Oshox22* was over-expressed in Zhonghua rice cultivar and transgenics were subjected to drought and salinity stresses (Zhang et al., 2012). In contrast, *Oshox22* mutant line was found to be tolerant to drought and salinity stresses at seedling stage (Zhang et al., 2012). Our findings can be correlated with other studies which have demonstrated the negative regulatory role of homeobox TFs, including OsBlHD1, Oshox22, and other TFs, such as OsAP2-39, OsbZIP52 and OsABI5 (Luo et al., 2005; Zou et al., 2008; Wan et al., 2011; Liu et al., 2012; Zhang et al., 2012).

The crucial function of ABA has already been delineated in abiotic stress responses in plants (Cutler et al., 2010; Fujita et al., 2011). In the last decade, ABA responsiveness of some HD-ZIP I family members in Arabidopsis and rice has been reported (Olsson et al., 2004; Valdés et al., 2012; Zhang et al., 2012). We have reported transgenic Arabidopsis seedlings over-expressing *OsHOX24* to be more sensitive than WT seedlings under exogenous ABA treatment (Bhattacharjee et al., 2016). Here also, we observed enhanced sensitivity of *OsHOX24* rice transgenics under exogenous ABA treatment at the seedling stage, which suggested an ABA-dependent mode of action of *OsHOX24* in stress responses. Stomatal closure is known to be a stress-adaptive mechanism in plants to prevent water loss (Osakabe et al., 2014). We found that *OsHOX24* rice transgenics possessed impaired ability of stomatal closure as compared to WT due to higher sensitivity to ABA, which suggested the role of *OsHOX24* in modulating abiotic stress responses in rice.

Varied abiotic stress responses in plants result from the interplay of events related to several metabolic pathways (Krasensky and Jonak, 2012; Obata and Fernie, 2012; Jain, 2013). The transcriptome analysis of rice transgenics over-expressing OsHOX24 and WT plants revealed downregulation of several genes related to cellular metabolism and macromolecular biosynthetic processes in the desiccation-stressed transgenics, which might contribute to the higher sensitivity of transgenics under DS. Notably, many of the differentially expressed genes were found to be involved in diverse biological pathways implicated directly or indirectly in abiotic stress responses. TFs are well known to orchestrate abiotic stress responses in plants (Nakashima et al., 2009; Todaka et al., 2015). We witnessed differential expression of greater number of TF-encoding genes (involved in metabolic processes, developmental processes, besides abiotic and hormone stimulus) in the transgenic line, which may be contributing to the susceptible nature of transgenics under DS. Transcriptome analysis of transgenic Arabidopsis over-expressing *OsHOX24* under control condition also revealed the existence of such diverse downstream target genes of OsHOX24 in our previous study (Bhattacharjee et al., 2016).

The role of plant hormones in abiotic stress responses has been well documented (Verma et al., 2016; Wani et al., 2016). Particularly, auxins have been delineated to have integral functions in plant development as well as abiotic stress responses (Jain and Khurana, 2009; Kazan, 2013; Sharma et al., 2015). We observed differential expression of various genes involved in hormonal signaling, namely auxin, ABA, ethylene and brassinosteroid signaling in the desiccation-stressed transgenics. Receptor-like kinases have been reported to act as major signaling components of plant development, in addition to abiotic stress responses (Marshall et al., 2012; Osakabe et al., 2013). We observed differential regulation of several such genes encoding for signaling molecules in the desiccation-stressed transgenics. In addition, elevated levels of secondary metabolites have also been reported in plants under abiotic stress conditions (Edreva et al., 2008; Krasensky and Jonak, 2012). Differential regulation of genes encoding for secondary metabolites was also witnessed in the transgenic line under DS. Alteration in the transcript level of these genes might change the physiology of transgenics leading to enhanced sensitivity. We speculate that even though, some of the stress-responsive genes were upregulated under stress condition, the collective effect of their gene products, i.e., protective molecules and secondary metabolites may not have been sufficient enough to elicit stress tolerance in transgenics. We detected relatively lesser extent of upregulation of many of the stress-responsive genes in the transgenics as compared to WT, which have been reported to play vital roles in plant abiotic stress responses and few among them have been found to elicit stress tolerance in transgenics (Jain et al., 2007; Xue et al., 2008; Cui et al., 2011; Ray et al., 2011; Chen et al., 2014; Sun et al., 2015). Interestingly, we found differential regulation of genes encoding for pathogenesis related (PR) proteins also in *OsHOX24* transgenic line. This suggested that OsHOX24 might be involved in mediating crosstalk between abiotic and biotic stress responses in rice.

## Conclusion

We demonstrated that over-expression of *OsHOX24* enhances the susceptibility of transgenic rice under different abiotic stress conditions at the seedling, mature, and reproductive stage of rice development. The over-expression of *OsHOX24* in rice leads to compromised growth at reproductive stage of development. Transcriptome analysis of transgenic rice and WT plants under DS and control condition led to identification of several plausible direct or indirect downstream targets of OsHOX24. These results can provide a new dimension to OsHOX24-mediated gene regulation. Altogether, the involvement of *OsHOX24* in abiotic stress responses besides plant development suggests its potential to be used as a promising candidate for crop improvement. In future, *OsHOX24* knock-down/knock-out transgenic lines can be raised to generate abiotic stress-tolerant rice plants.

## Author Contributions

MJ conceived and supervised the whole study. AB and RS performed all the experiments and analyzed data. AB wrote the manuscript. MJ participated in data analysis and writing of the manuscript.

## Conflict of Interest Statement

The authors declare that the research was conducted in the absence of any commercial or financial relationships that could be construed as a potential conflict of interest.

## Footnotes

1 http://mapman.gabipd.org/web/guest

2 http://bioinfogp.cnb.csic.es/tools/venny/index.html

## References

[B1] Agalou A. Purwantomo S. Övernäs E. Johannesson H. Zhu X. Estiati A. (2008). A genome-wide survey of HD-Zip genes in rice and analysis of drought-responsive family members. *Plant Mol. Biol.* 66 87–103. 10.1007/s11103-007-9255-7 17999151

[B2] Alavilli H. Awasthi J. P. Rout G. R. Sahoo L. Lee B. H. Panda S. K. (2016). Overexpression of a barley aquaporin gene, *HvPIP2;5* confers salt and osmotic stress tolerance in yeast and plants. *Front. Plant Sci.* 7:1566 10.3389/fpls.2016.01566 PMC5073208 27818670

[B3] Ariel F. Diet A. Verdenaud M. Gruber V. Frugier F. Chan R. (2010). Environmental regulation of lateral root emergence in *Medicago truncatula* requires the HD-Zip I transcription factor HB1. *Plant Cell* 22 2171–2183. 10.1105/tpc.110.074823 20675575 PMC2929095

[B4] Ariel F. D. Manavella P. A. Dezar C. A. Chan R. L. (2007). The true story of the HD-Zip family. *Trends Plant Sci.* 12 419–426. 10.1016/j.tplants.2007.08.003 17698401

[B5] Bhattacharjee A. Ghangal R. Garg R. Jain M. (2015). Genome-wide analysis of homeobox gene family in legumes: identification, gene duplication and expression profiling. *PLoS ONE* 10:e0119198 10.1371/journal.pone.0119198 PMC4352023 25745864

[B6] Bhattacharjee A. Jain M. (2013). “Homeobox genes as potential candidates for crop improvement under abiotic stress,” in *Plant Acclimation to Environmental Stress*, eds Tuteja N. Gill S. S. (New York, NY: Springer Science+Business Media), 163–176. 10.1007/978-1-4614-5001-6_7

[B7] Bhattacharjee A. Khurana J. P. Jain M. (2016). Characterization of rice homeobox genes, *OsHOX22* and *OsHOX24*, and over-expression of *OsHOX24* in transgenic *Arabidopsis* suggest their role in abiotic stress response. *Front. Plant Sci.* 7:627 10.3389/fpls.2016.00627 PMC4862318 27242831

[B8] Chen X. Wang Y. Lv B. Li J. Luo L. Lu S. (2014). The NAC family transcription factor OsNAP confers abiotic stress response through the ABA pathway. *Plant Cell Physiol.* 55 604–619. 10.1093/pcp/pct204 24399239

[B9] Chew W. Hrmova M. Lopato S. (2013). Role of homeodomain leucine zipper (HD-Zip) IV transcription factors in plant development and plant protection from deleterious environmental factors. *Int. J. Mol. Sci.* 14 8122–8147. 10.3390/ijms14048122 23584027 PMC3645734

[B10] Cui M. Zhang W. Zhang Q. Xu Z. Zhu Z. Duan F. (2011). Induced over-expression of the transcription factor OsDREB2A improves drought tolerance in rice. *Plant Physiol. Biochem.* 49 1384–1391. 10.1016/j.plaphy.2011.09.012 22078375

[B11] Cutler S. R. Rodriguez P. L. Finkelstein R. R. Abrams S. R. (2010). Abscisic acid: emergence of a core signaling network. *Annu. Rev. Plant Biol.* 61 651–679. 10.1146/annurev-arplant-042809-112122 20192755

[B12] Dansana P. K. Kothari K. S. Vij S. Tyagi A. K. (2014). OsiSAP1 overexpression improves water-deficit stress tolerance in transgenic rice by affecting expression of endogenous stress-related genes. *Plant Cell Rep.* 33 1425–1440. 10.1007/s00299-014-1626-3 24965356

[B13] Deng X. Phillips J. Brautigam A. Engstrom P. Johannesson H. Ouwerkerk P. B. F. (2006). A homeodomain leucine zipper gene from *Craterostigma plantagineum* regulates abscisic acid responsive gene expression and physiological responses. *Plant Mol. Biol.* 61 469–489. 10.1007/s11103-006-0023-x 16830180

[B14] Deng X. Phillips J. Meijer A. H. Salamini F. Bartels D. (2002). Characterization of five novel dehydration-responsive homeodomain leucine zipper genes from the resurrection plant *Craterostigma plantagineum*. *Plant Mol. Biol.* 49 601–610. 10.1023/A:1015501205303 12081368

[B15] Edreva A. Velikova V. Tsonev T. Dagnon S. Gurel A. Aktas L. (2008). Stress-protective role of secondary metabolites: diversity of functions and mechanisms. *Gen. Appl. Plant Physiol.* 34 67–78.

[B16] Fujita Y. Fujita M. Shinozaki K. Yamaguchi-Shinozaki K. (2011). ABA-mediated transcriptional regulation in response to osmotic stress in plants. *J. Plant Res.* 124 509–525. 10.1007/s10265-011-0412-3 21416314

[B17] Golldack D. Luking I. Yang O. (2011). Plant tolerance to drought and salinity: stress regulating transcription factors and their functional significance in the cellular transcriptional network. *Plant Cell Rep.* 30 1383–1391. 10.1007/s00299-011-1068-0 21476089

[B18] Jain M. (2013). Emerging role of metabolic pathways in abiotic stress tolerance. *J. Plant Biochem. Physiol.* 1:108 10.4172/2329-9029.1000108

[B19] Jain M. Khurana J. P. (2009). Transcript profiling reveals diverse roles of auxin-responsive genes during reproductive development and abiotic stress in rice. *FEBS J.* 276 3148–3162. 10.1111/j.1742-4658.2009.07033.x 19490115

[B20] Jain M. Nijhawan A. Arora R. Agarwal P. Ray S. Sharma P. (2007). F-box proteins in rice. Genome-wide analysis, classification, temporal and spatial gene expression during panicle and seed development, and regulation by light and abiotic stress. *Plant Physiol.* 143 1467–1483. 10.1104/pp.106.091900 17293439 PMC1851844

[B21] Jain M. Tyagi A. K. Khurana J. P. (2006). Over expression of putative topoisomerase 6 genes from rice confers stress tolerance in transgenic *Arabidopsis* plants. *FEBS J.* 273 5245–5260. 10.1111/j.1742-4658.2006.05518.x 17116242

[B22] Jain M. Tyagi A. K. Khurana J. P. (2008). Genome-wide identification, classification, evolutionary expansion and expression analyses of homeobox genes in rice. *FEBS J.* 275 2845–2861. 10.1111/j.1742-4658.2008.06424.x 18430022

[B23] Jang J. Y. Lee S. H. Rhee J. Y. Chung G. C. Ahn S. J. Kang H. (2007). Transgenic *Arabidopsis* and tobacco plants overexpressing an aquaporin respond differently to various abiotic stresses. *Plant Mol. Biol.* 64 621–632. 10.1007/s11103-007-9181-8 17522953

[B24] Johannesson H. Wang Y. Engström P. (2001). DNA-binding and dimerization preferences of *Arabidopsis* homeodomain-leucine zipper transcription factors in vitro. *Plant Mol. Biol.* 45 63–73. 10.1023/A:1006423324025 11247607

[B25] Kazan K. (2013). Auxin and the integration of environmental signals into plant root development. *Ann. Bot.* 112 1655–1665. 10.1093/aob/mct229 24136877 PMC3838554

[B26] Krasensky J. Jonak C. (2012). Drought, salt, and temperature stress-induced metabolic rearrangements and regulatory networks. *J. Exp. Bot.* 63 1593–1608. 10.1093/jxb/err460 22291134 PMC4359903

[B27] Liu C. Wu Y. Wang X. (2012). bZIP transcription factor *OsbZIP52*/*RISBZ5*: a potential negative regulator of cold and drought stress response in rice. *Planta* 235 1157–1169. 10.1007/s00425-011-1564-z 22189955

[B28] Luo H. Song F. Zheng Z. (2005). Over-expression in transgenic tobacco reveals different roles for the rice homeodomain gene *OsBIHD1* in biotic and abiotic stress responses. *J. Exp. Bot.* 56 2673–2682. 10.1093/jxb/eri260 16105854

[B29] Manavella P. A. Arce A. L. Dezar C. A. Bitton F. Renou J. P. Crespi M. (2006). Cross-talk between ethylene and drought signaling pathways is mediated by the sunflower Hahb-4 transcription factor. *Plant J.* 48 125–137. 10.1111/j.1365-313X.2006.02865.x 16972869

[B30] Marshall A. Aalen R. B. Audenaert D. Beeckman T. Broadley M. R. Butenko M. A. (2012). Tackling drought stress: receptor-like kinases present new approaches. *Plant Cell* 24 2262–2278. 10.1105/tpc.112.096677 22693282 PMC3406892

[B31] Meijer A. H. de Kam R. J. D’Erfurth I. Shen W. Hoge J. H. (2000). HD-Zip proteins of families I and II from rice: interactions and functional properties. *Mol. Gen. Genet.* 263 12–21. 10.1007/PL00008671 10732669

[B32] Mukherjee K. Brocchieri L. Bürglin T. R. (2009). A comprehensive classification and evolutionary analysis of plant homeobox genes. *Mol. Biol. Evol.* 26 2775–2794. 10.1093/molbev/msp201 19734295 PMC2775110

[B33] Nakashima K. Ito Y. Yamaguchi-Shinozaki K. (2009). Transcriptional regulatory networks in response to abiotic stresses in *Arabidopsis* and grasses. *Plant Physiol.* 149 88–95. 10.1104/pp.108.129791 19126699 PMC2613698

[B34] Obata T. Fernie A. R. (2012). The use of metabolomics to dissect plant responses to abiotic stresses. *Cell. Mol. Life Sci.* 69 3225–3243. 10.1007/s00018-012-1091-5 22885821 PMC3437017

[B35] Olsson A. S. B. Engström P. Söderman E. (2004). The homeobox genes ATHB12 and ATHB7 encode potential regulators of growth in response to water deficit in *Arabidopsis*. *Plant Mol. Biol.* 55 663–677. 10.1007/s11103-004-1581-4 15604708

[B36] Osakabe Y. Osakabe K. Shinozaki K. Tran L. S. P. (2014). Response of plants to water stress. *Front. Plant Sci.* 5:86 10.3389/fpls.2014.00086 PMC3952189 24659993

[B37] Osakabe Y. Yamaguchi-Shinozaki K. Shinozaki K. Tran L. (2013). Sensing the environment: key roles of membrane-localized kinases in plant perception and response to abiotic stress. *J. Exp. Bot.* 64 445–458. 10.1093/jxb/ers354 23307915

[B38] Palena C. M. Gonzalez D. H. Chan R. L. (1999). A monomer-dimer equilibrium modulates the interaction of the sunflower homeodomain leucine-zipper protein Hahb-4 with DNA. *Biochem. J.* 341 81–87. 10.1042/0264-6021:3410081 10377247 PMC1220332

[B39] Ray S. Dansana P. K. Giri J. Deveshwar P. Arora R. Agarwal P. (2011). Modulation of transcription factor and metabolic pathway genes in response to water-deficit stress in rice. *Funct. Integr. Genomics* 11 157–178. 10.1007/s10142-010-0187-y 20821243

[B40] Sessa G. Morelli G. Ruberti I. (1997). DNA-binding specificity of the homeodomain-leucine zipper domain. *J. Mol. Biol.* 274 303–309. 10.1006/jmbi.1997.1408 9405140

[B41] Sharma E. Sharma R. Borah P. Jain M. Khurana J. P. (2015). “Emerging roles of auxin in abiotic stress responses,” in *Elucidation of Abiotic Stress Signaling in Plants*, ed. Pandey G. K. (New York, NY: Springer+Business Media), 299–328. 10.1007/978-1-4939-2211-6_11

[B42] Sharma R. Priya P. Jain M. (2013). Modified expression of an auxin-responsive rice CC-type glutaredoxin gene affects multiple abiotic stress responses. *Planta* 238 871–884. 10.1007/s00425-013-1940-y 23918184

[B43] Song S. Chen Y. Zhao M. Zhang W. H. (2012). A novel *Medicago truncatula* HD-Zip gene, MtHB2, is involved in abiotic stress responses. *Environ. Exp. Bot.* 80 1–9. 10.1016/j.envexpbot.2012.02.001

[B44] Sun L. Huang L. Hong Y. Zhang H. Song F. Li D. (2015). Comprehensive analysis suggests overlapping expression of rice ONAC transcription factors in abiotic and biotic stress responses. *Int. J. Mol. Sci.* 16 4306–4326. 10.3390/ijms16024306 25690040 PMC4346958

[B45] Todaka D. Shinozaki K. Yamaguchi-Shinozaki K. (2015). Recent advances in the dissection of drought-stress regulatory networks and strategies for development of drought-tolerant transgenic rice plants. *Front. Plant Sci.* 6:84 10.3389/fpls.2015.00084 PMC4332304 25741357

[B46] Tran L. S. P. Nakashima K. Sakuma Y. Osakabe Y. Qin F. Simpson S. D. (2007). Co-expression of the stress-inducible zinc finger homeodomain ZFHD1 and NAC transcription factors enhances expression of the *ERD1* gene in *Arabidopsis*. *Plant J.* 49 46–63. 10.1111/j.1365-313X.2006.02932.x 17233795

[B47] Turchi L. Baima S. Morelli G. Ruberti I. (2015). Interplay of HD-Zip II and III transcription factors in auxin-regulated plant development. *J. Exp. Bot.* 66 5043–5053. 10.1093/jxb/erv174 25911742

[B48] Urano K. Kurihara Y. Seki M. Shinozaki K. (2010). ‘Omics’ analyses of regulatory networks in plant abiotic stress responses. *Curr. Opin. Plant Biol.* 13 132–138. 10.1016/j.pbi.2009.12.006 20080055

[B49] Valdés A. E. Övernäs E. Johansson H. Rada-Iglesias A. Engström P. (2012). The homeodomain-leucine zipper (HD-Zip) class I transcription factors ATHB7 and ATHB12 modulate abscisic acid signalling by regulating protein phosphatase 2C and abscisic acid receptor gene activities. *Plant Mol. Biol.* 80 405–418. 10.1007/s11103-012-9956-4 22968620

[B50] Verma V. Ravindran P. Kumar P. P. (2016). Plant hormone-mediated regulation of stress responses. *BMC Plant Biol.* 16:86 10.1186/s12870-016-0771-y PMC4831116 27079791

[B51] Wan L. Zhang J. Zhang H. Zhang Z. Quan R. Zhou S. (2011). Transcriptional activation of OsDERF1 in OsERF3 and OsAP2-39 negatively modulates ethylene synthesis and drought tolerance in rice. *PLoS ONE* 6:e25216 10.1371/journal.pone.0025216 PMC3180291 21966459

[B52] Wang H. Wang H. Shao H. Tang X. (2016). Recent advances in utilizing transcription factors to improve plant abiotic stress tolerance by transgenic technology. *Front. Plant Sci.* 7:67 10.3389/fpls.2016.00067 PMC4746321 26904044

[B53] Wani S. H. Kumar V. Shriram V. Sah S. K. (2016). Phytohormones and their metabolic engineering for abiotic stress tolerance in crop plants. *Crop J.* 4 162–176. 10.1016/j.cj.2016.01.010

[B54] Wu Q. Li D. Li D. Liu X. Zhao X. Li X. (2015). Overexpression of OsDof12 affects plant architecture in rice (*Oryza sativa* L.). *Front. Plant Sci.* 6:833 10.3389/fpls.2015.00833 PMC4597119 26500670

[B55] Xue T. Wang D. Zhang S. Ehlting J. Ni F. Jakab S. (2008). Genome-wide and expression analysis of protein phosphatase 2C in rice and Arabidopsis. *BMC Genomics* 9:550 10.1186/1471-2164-9-550 PMC2612031 19021904

[B56] Yu H. Chen X. Hong Y. Y. Wang Y. Xu P. Ke S. D. (2008). Activated expression of an *Arabidopsis* HD-START protein confers drought tolerance with improved root system and reduced stomatal density. *Plant Cell* 20 1134–1151. 10.1105/tpc.108.058263 18451323 PMC2390749

[B57] Yu L. Chen X. Wang Z. Wang S. Wang Y. Zhu Q. (2013). Arabidopsis *Enhanced Drought Tolerance1*/*HOMEODOMAIN GLABROUS11* confers drought tolerance in transgenic rice without yield penalty. *Plant Physiol.* 162 1378–1391. 10.1104/pp.113.217596 23735506 PMC3707532

[B58] Zhang S. Haider I. Kohlen W. Jiang L. Bouwmeester H. Meijer A. H. (2012). Function of the HD-Zip I gene *OsHox22* in ABA-mediated drought and salt tolerances in rice. *Plant Mol. Biol.* 80 571–585. 10.1007/s11103-012-9967-1 23109182

[B59] Zhao Y. Zhou Y. Jiang H. Li X. Gan D. Peng X. (2011). Systematic analysis of sequences and expression patterns of drought-responsive members of the HD-Zip gene family in maize. *PLoS ONE* 6:e28488 10.1371/journal.pone.0028488 PMC3229603 22164299

[B60] Zheng K. Tian H. Hu Q. Guo H. Yang L. Cai L. (2016). Ectopic expression of R3 MYB transcription factor gene *OsTCL1* in Arabidopsis, but not rice, affects trichome and root hair formation. *Sci. Rep.* 6:19254 10.1038/srep19254 PMC4725938 26758286

[B61] Zou M. Guan Y. Ren H. Zhang F. Chen F. (2008). A bZIP transcription factor, OsABI5, is involved in rice fertility and stress tolerance. *Plant Mol. Biol.* 66 675–683. 10.1007/s11103-008-9298-4 18236009

