# Supplementary material for: Over-Expression of OsHOX24 Confers Enhanced Susceptibility to Abiotic Stresses in Transgenic Rice via Modulating Stress-Responsive Gene Expression
Source: Front Plant Sci. 2017 Apr 21;8:628. doi: 10.3389/fpls.2017.00628 (PMC5399076; doi:10.3389/fpls.2017.00628)
Supplement: Supplementary file 1 [file Data_Sheet_1.pdf]

Supplementary Material:

Figure. S1 Phenotype of *OsHOX24* over-expression rice transgenics under control condition at seedling stage. Growth phenotype of seven-day-old rice seedlings (A) and five-week-old (B) wild-type (WT) and *OsHOX24* rice transgenic lines, H1, H49 and H74 under control condition are shown.

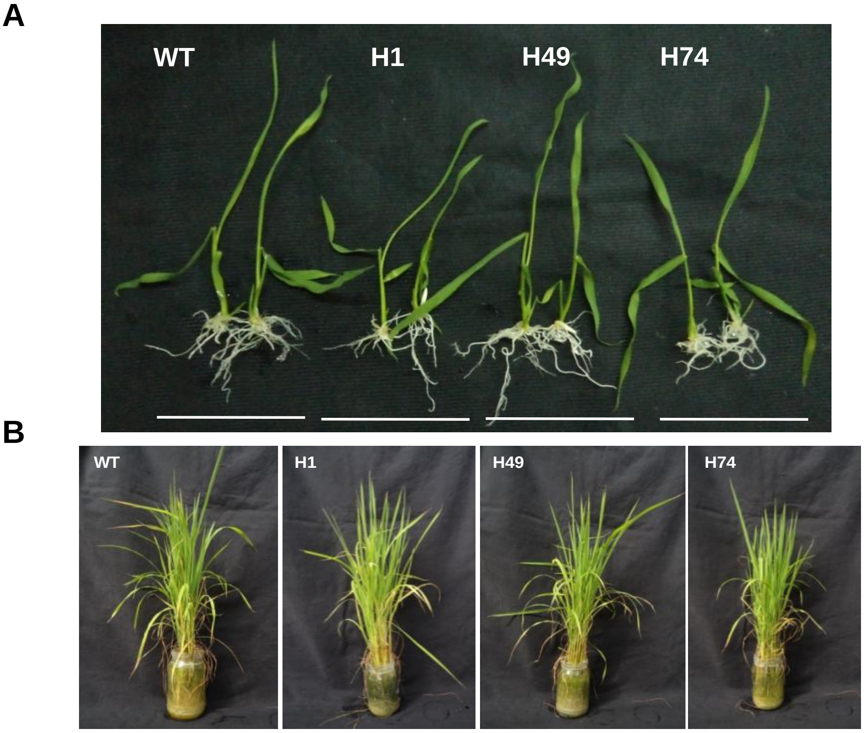

Figure. S2 Phenotype of *OsHOX24* over-expression rice transgenics and wild-type (WT) plants at reproductive stage. (A) Growth phenotype of 135-day-old WT and *OsHOX24* rice transgenic lines, H1, H49 and H74 under control condition are shown. The shoot length (B), flag-leaf area (C), number of tillers (D) and number of panicles (E) per plant for *UBQ::OsHOX24* over-expression rice transgenics and WT plants were recorded. The experiments were conducted in at least three independent biological replicates. Values are mean from one representative biological replicate, where readings from at least ten independent plants were recorded for the transgenic lines under control condition. Error bars indicate SE. Data points marked with asterisk ("**" $P \leq 0.01$, "*" $P \leq 0.05$) indicate statistically significant difference between WT and transgenic plants.

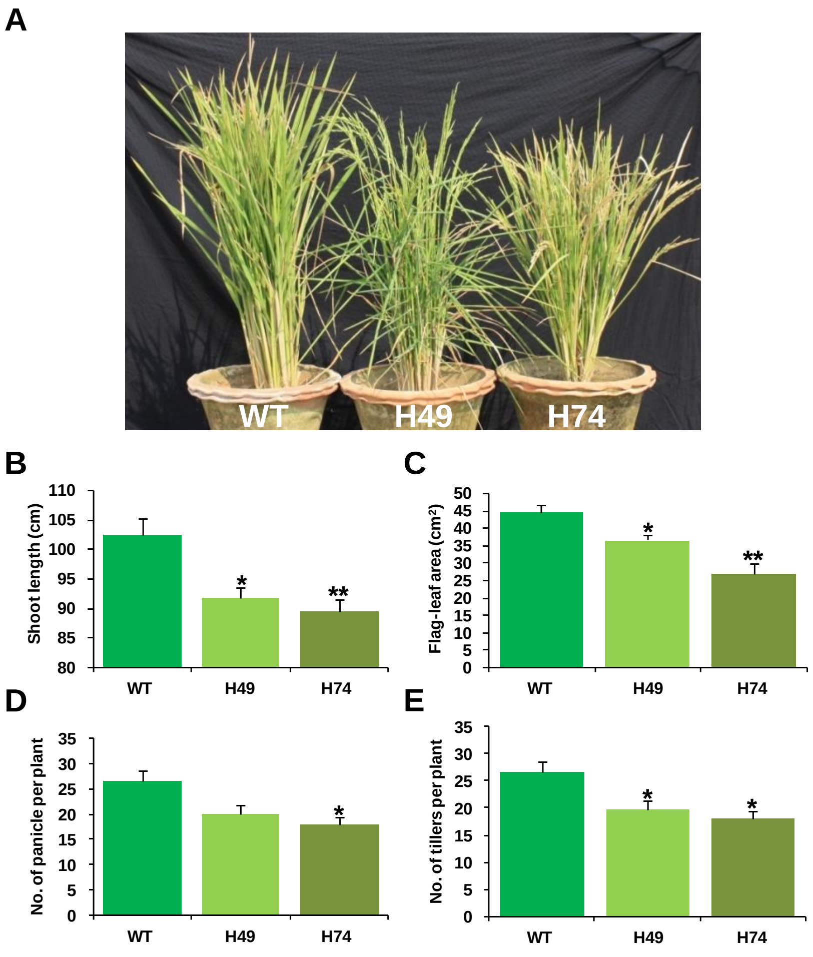

Figure. S3 Water loss of *OsHOX24* over-expression rice as compared to wild-type (WT) plants at mature and reproductive stages. The percentage fresh weight of detached leaves of WT and H49 transgenic line measured at 30 min interval for 210 min at mature (A) and reproductive (B) stages are shown. The experiments were conducted in at least two independent biological replicates. Values are mean from two representative biological replicates, where readings of three independent leaves from plants (N=3) were recorded during air drying. Error bars indicate SE.

A

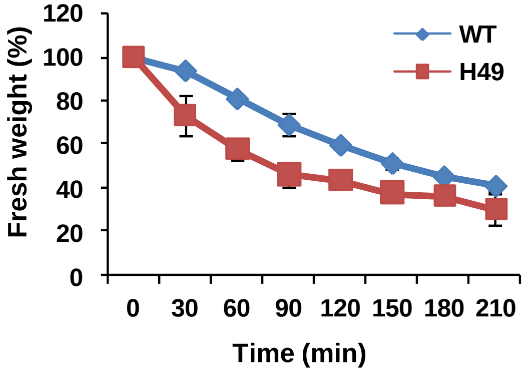

B

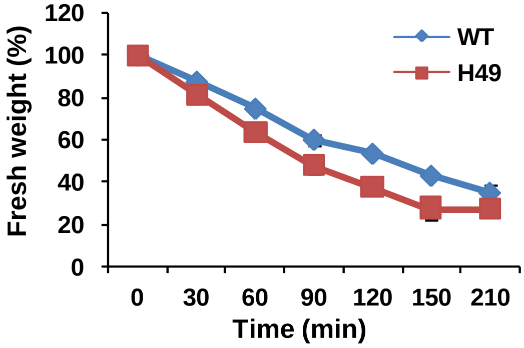

Figure. S4 Phenotype of *OsHOX24* over-expression rice transgenics as compared to wild-type (WT) plants under desiccation stress at mature stage. Leaves of two-month-old WT and *UBQ*::*OsHOX24* rice transgenic lines (H49 and H74) were incubated in PEG 6000 solution (20%) for three days. The bar graph on the right side shows the relative chlorophyll content percentage of WT and transgenic leaves after desiccation stress treatment. Values are mean from two independent experiments. Error bars indicate SE.

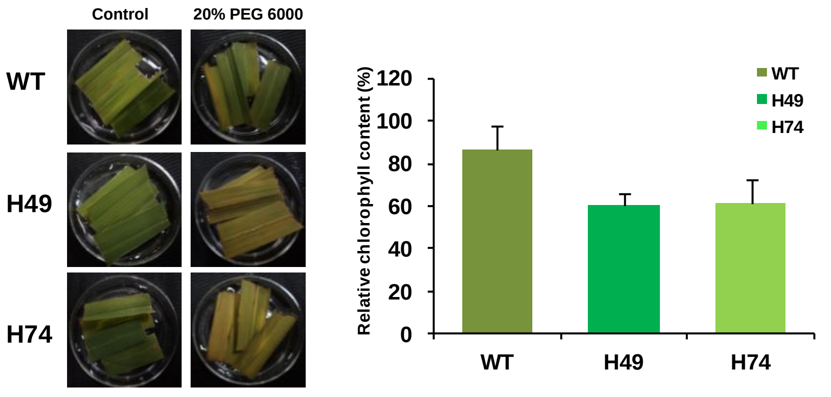

Figure. S5 Differential expression of transcription factors (TFs) in *OsHOX24* transgenic and wild-type (WT) plants under control and desiccation stress (DS). Heat-map represents expression profiles of 220 differentially expressed TF-encoding genes in H49_WT_CT (differential expression between WT and transgenic line under control condition), WT_DS (differential expression in WT under desiccation and control condition) and H49_DS (differential expression in transgenic line under desiccation and control condition). The color scale representing average log signal values is shown below. The TF family has been indicated on the right.

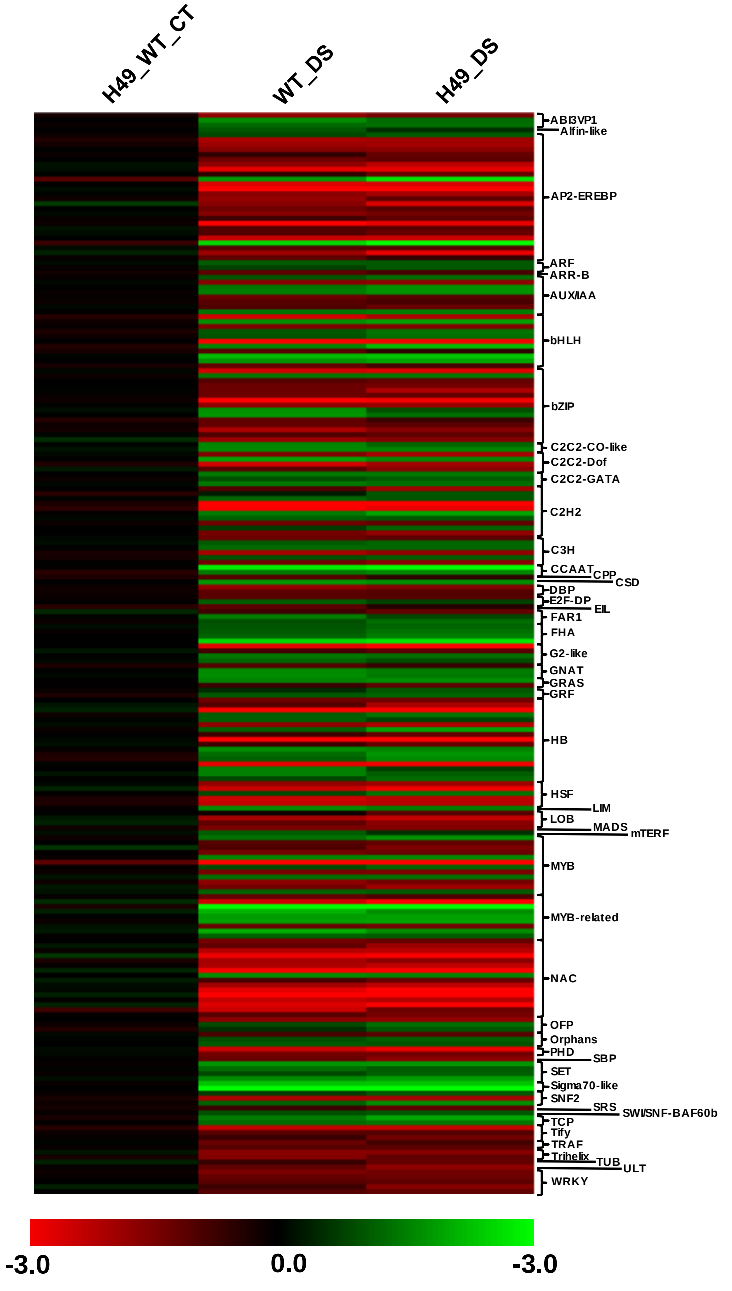

Figure. S6 Overview of metabolic pathways involving differentially expressed transcripts in *OsHOX24* transgenics under stress conditions. Metabolic pathway overview (from Mapman) for the differentially expressed transcripts in *OsHOX24* transgenics under desiccation stress (DS) condition are shown. Red, up-regulated transcripts and blue, down-regulated transcripts.

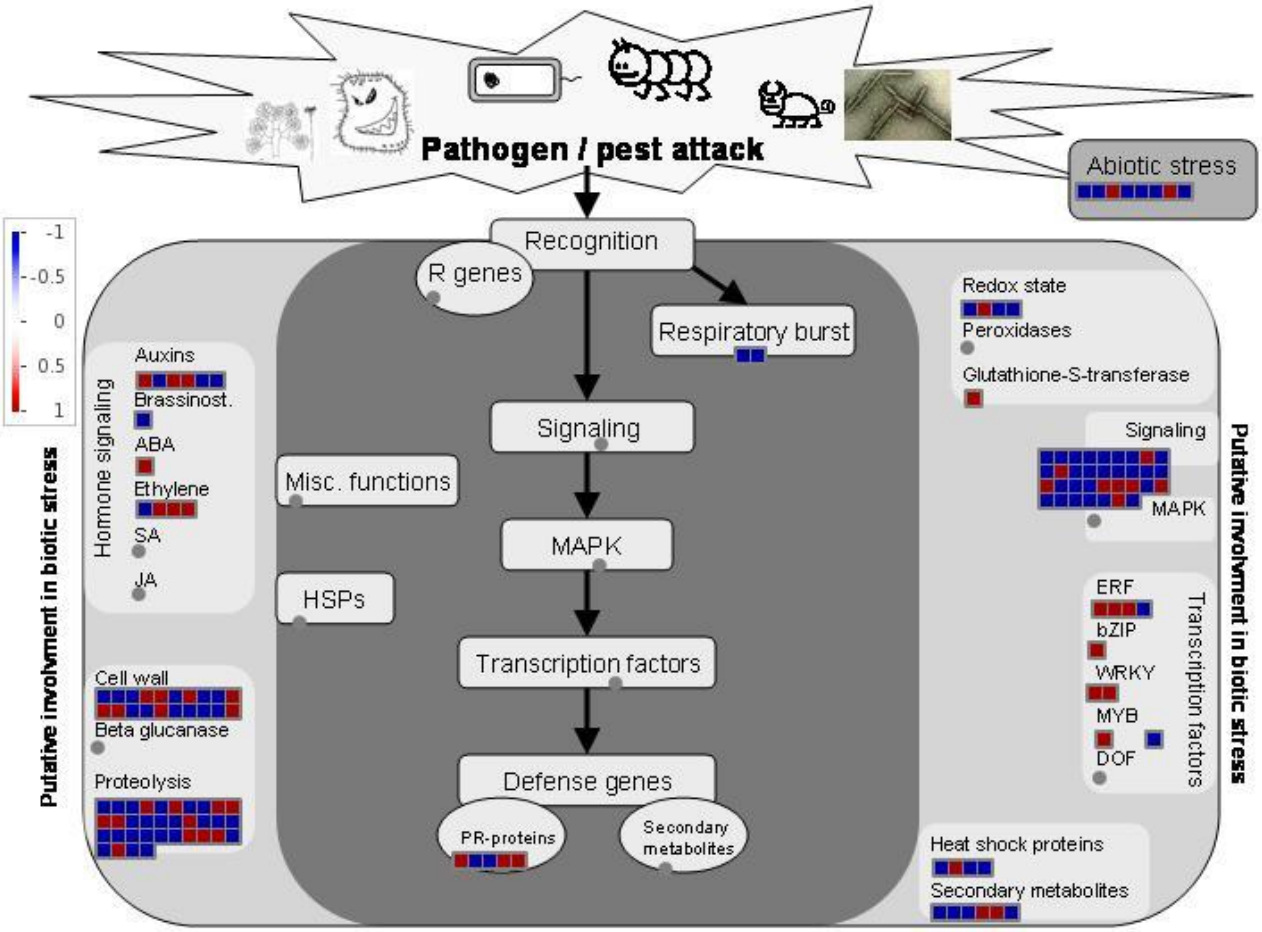

Figure. S7 Real time PCR validation of microarray results. Comparison of expression profiles of selected differentially expressed stress-responsive rice genes in wild-type (WT) and *OsHOX24* over-expression transgenic line (H49) under desiccation stress are shown. The expression levels were calculated relative to the gene expression of WT and transgenic seedlings under control condition.

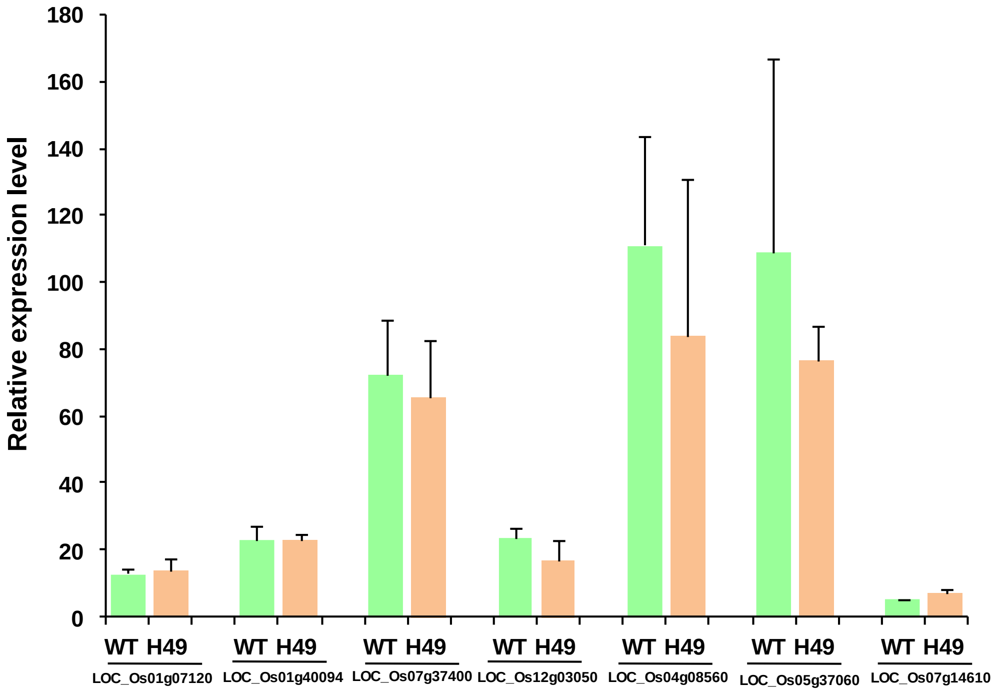

Table S1 Primers used for real-time PCR and cloning experiments

Primer Name	Sequence of Primer	Purpose
OsHOX24_R_FP	5′ TACGAGTACGACCATCACCTAGACTAC 3′	For real-time PCR analysis
OsHOX24_R_RP	5′ TGGCCATATCTCCCACAGATC 3′	
UBQ5_R_FP	5′ ACCACTTCGACCGCCACTACT 3′	
UBQ5_R_RP	5′ ACGCCTAAGCCTGCTGGTT 3′	
LOC_Os01g07120 RT FP	5′ GGGTCCAAGAAAGGGTGCAT 3′	For real-time PCR validation of microarray data analysis
LOC_Os01g07120 RT RP	5′ GCGGTAAGCACAATTTGAATTTT 3′	
LOC_Os01g40094 RT FP	5′ CGGCTGTAGTCGCTGTGATC 3′	
LOC_Os01g40094 RT RP	5′ GCCCGTGAGTCTCCACAATT 3′	
LOC_Os07g37400 RT FP	5′ TGCTCGAATCACTGCCTCAA 3′	
LOC_Os07g37400 RT RP	5′ GGCCTCAGGTCACTGTGGTT 3′	
LOC_Os12g03050 RT FP	5′ GACGGACGCTGAGGTAATCTTAAG 3′	
LOC_Os12g03050 RT RP	5′ TGGGCAAGGAGGTGAAGCTA 3′	
LOC_Os04g08560 RT FP	5′ CGCGAGCATTAGGAGACAAGT 3′	
LOC_Os04g08560 RT RP	5′ GTTATGGTGATGTCTGGCTCACA 3′	
LOC_Os05g37060 RT FP	5′ GGCTATCTAAGCACCGGCATT 3′	
LOC_Os05g37060 RT RP	5′ CGCGTATACTTGCTGTGCTCTT 3′	
LOC_Os07g14610 RT FP	5′ TGAAGAGGATCAAAGAGATAGTGGAA 3′	
LOC_Os07g14610 RT RP	5′ GCATCGACTCCTCCATGAAGTC 3′	
pB4NU-OsHOX24 FP	5′ ATAGGATCCATGGAGAGCGACTG 3′	For cloning in modified pCAMBIA1301 vector
pB4NU-OsHOX24 RP	5′ ATTGGTACCTCATGCTACTGCGTT 3′	

Table S2 List of differentially expressed genes in the rice transgenic (H49) and wild-type (WT) under control and desiccation stress (DS) (Available as a separate excel file). The values in the table represent significant fold change values ≥2 fold (with corrected *P*-value ≤0.05)
